# Supplementary material for: Too many yet too few caesarean section deliveries in Bangladesh: Evidence from Bangladesh Demographic and Health Surveys data
Source: PLOS Glob Public Health. 2022 Feb 2;2(2):e0000091. doi: 10.1371/journal.pgph.0000091 (PMC10022004; doi:10.1371/journal.pgph.0000091)
Supplement: Attachment — Submitted filename: Response to editor and reviwers.docx [file pgph.0000091.s002.docx]

**Editor’s and Reviewers’ comments and author’s responses**

| **Academic editor’s comment** | **Authors’ response** | **Reference** |
| --- | --- | --- |
| Dear Authors: Please answer/include each one of the reviewers' suggestions. Also it is needed that you review the following aspects: | We have revised the manuscript considering the reviewers’ comments. A point-by-point response to each of the reviewers’ comments are also provided here. | N/A |
| 1. The title does not include the design, as Strobe suggests. | We have revised the title as follows-  *Too many yet too few caesarean section deliveries in Bangladesh: evidence from the Bangladesh Demographic and Health Surveys Data* | Page 1 |
| 2. The sample size of each one of the rounds should be included in the methods, and each one of the tables in which they are used. | The sample size for each survey has added in the newly added figure, Figure 1. We have now referred this figure in the relevant sentence of the method section. It reads-  *Total of 27,093 women data, as presented in Figure 1, were extracted from these five rounds of BDHS for analysis.*  Whereas the newly added figure is presented in Page 5. | Page 6 |
| 3. The adequate use of sampling weights when the analysis was performed, should be included in the methods section | We were considered sampling weights during our initial analysis which we have now mentioned in the statistical analysis section under the Methods. It reads-  *Sampling weights were considered in all analyses.* | Page 7 |
| 4. Given that the original sample size was not estimated to find prevalence at facility and division levels, a paragraph should be included to explain how authors handled this limitation for the analysis and for the discussion of their results. | We appreciate editor for this important comment. However, as part of the broad revision, division related sentences have been deleted. Therefore, such focus on divisional level sample size is unnecessary now. | NA |
| 5. In figure 1, confidence intervals of values should be included in the figure | We have added the confidence intervals of values in Figure 2 (old figure number 1). Please see the revised figure. | NA |
| 6. In Table 2. a definition of "institutional", "population caesarean" and "institutional caesarean", should be included as a table-note | We have now added table footnotes in table 2 and provided definition for each term. It reads-  *Note: ^1^Percentage of total delivery occurred in any forms of healthcare facilities; ^2^Percentage of total cesarean delivery without considering delivery places, ^3^Percentage of total cesarean delivery among institutional delivery.* | NA |
| 7. In Table 3, Interaction of working status and place of residence values for 2004 should be double checked | Thanks to bring this in our attention. We have checked this and corrected the significance level. | NA |
| 8. In Table 3 add table-notes to make it clearer | We are not sure about the quarry here, however, we think it was for missing significance level that we have added now as table footnote. Please see the revised version. |  |
| 9. In Figure 3 y-labels used are not clear. It is not clear how are they different. If rural place and lower wealth quintile are common among them, these characteristics could be included in the title of the figure. The y-label should use the additional disadvantage that is different. Please use understandable labels (the label of the figure 3b label is not understandable) | We have revised this figure and edited figure’s legend to make it understandable to all. Please see Figure 4 (old figure number 3). |  |
| 10. Ethics statement should be added to the manuscript | We have added an ethics statement section which now reads-  *The BDHSs data collection methods and procedure were reviewed and approved by the institutional review board of ICF and the National Research Ethics Committee of the Bangladesh Medical Research Council. They also provided ethical approval of this survey. Informed consent was obtained from all participants during surveys and data were released in deidentified form. Therefore, no additional ethical approval was required for this study.* | Page 7 |
| **Reviewer#1** |  |  |
| The article “Too many yet too few caesarean section deliveries in Bangladesh: an ongoing public health challenge to improve maternal and child health” is a good effort from the authors to address a very important health issue of the developing world. The authors should address following suggestions to improve their manuscript |  |  |
| Abstract |  |  |
| It is too lengthy, especially the background section, kindly reduce the number of words so that readers can have a better understanding. | We have revised the abstract to reduce its length. The background section of the abstract has also revised. The revised background section now reads-  *Caesarean section (CS) use is rising rapidly in Bangladesh, though lack of CS use remains common among disadvantage women. This increases risks of long-term obstetric complications as well as maternal and child deaths among disadvantage women. We aimed to determine the interaction effects of women’s disadvantage characteristics on CS use in Bangladesh.* | Page 1 |
| Introduction |  |  |
| Kindly keep the format of citations uniform (space between the citation and the last word). “Only 10% of the total countries worldwide have the CS rate 10-15%[3],” | Thanks to bring this formatting issue in our attention. We have now revised this throughout the manuscript. | NA |
| Kindly cite articles from the developing countries that are on the same topic (A Cross-Sectional Study to Assess the Frequency and Risk Factors Associated with Cesarean Section in Southern Punjab, Pakistan. Int. J. Environ. Res. Public Health 2021, 18, 8812.[https://doi.org/10.3390/ijerph18168812](https://protect-au.mimecast.com/s/uFvZCVARWBc9nl6LuQD62v?domain=doi.org)). | We have now cited the article you suggested along with other relevant articles. | NA |
| **Methods** |  |  |
| Was there any ethical approval taken for the study? Or it was not required? Kindly mention it clearly. | The issue has been addressed to address an editor’s comment. A section, named “Ethical Statement”, has added and all relevant details are provided. The added section reads-  *The BDHSs data collection methods and procedure were reviewed and approved by the institutional review board of ICF and the National Research Ethics Committee of the Bangladesh Medical Research Council. They also provided ethical approval of this survey. Informed consent was obtained from all participants during surveys and data were released in deidentified form. Therefore, no additional ethical approval was required for this study.* | Page 7 |
| **Results** |  |  |
| Table 1, Kindly add in the method section how exposure to the mass media was categorized? | We have added details of this variable creation procedure. Please see the revised version. | Page 5-6 |
| How were the participants categorized into different wealth status? | We have added details of this variable creation procedure. Please see the revised version. | Page 5-6 |
| Figure 2: kindly provide y-axis on every subset separately as it is hard to follow in its current form. | As suggested, we have added y-axis on every subset separately. | NA |
| Figure 3: the title is hard to follow kindly rephrase it to make it easy for the readers. | We have revised this figure to make it understandable. Please see the revised figure. | NA |
| **Reviewer#2** |  |  |
| The article is very interesting and addresses a timely issue. It addresses the challenges of maternal mortality by addressing the issue of inequity of care in the delivery of maternal services. |  |  |
| **Title** |  |  |
| The study design must be specified in the title | We have added the study design in the title. The revised title reads-  *Too many yet too few caesarean section deliveries in Bangladesh: evidence from the Bangladesh Demographic and Health Surveys Data* | Page 1 |
| **Key words** |  |  |
| The keywords must be reviewed : choose between double burden and triple burden : using both are confusing. | We have revised the keywords and deleted a key words: double burden. | Page 1 |
| **Introduction** |  |  |
| Bangladesh is one of the LMICs, so it is unnecessary to single it out in paragraph 4 of the introduction. The reference to anemia and nutritional problems at this point is not justified as it is not relevant to the topic. The authors should review the relevance of this sentence in the introduction. The authors specify that there were differences in the use of CS in certain geographic regions. Can they show the specifics of the regions in question? | We have revised the relevant sections to address these issues. The sentence related to the anemia and nutritional disorder has deleted and the regions in Bangladesh where CS use is high have specified. The edited section reads-  *The CS use in LMICs is generally linked to the level of development including women’s education, fertility level, wealth, and contribution of the private healthcare facilities in providing CS [1, 2]. Consequently, a group of women could not access this service because of financial hardship whereas another group use this service without medical necessity — an indication of the double burden of CS [2]. Moreover, along with financial capacity, geographical variation of availability of this service as well as geographical hardship to access services, such as poor transportation, could also play a significant role in differencing the CS use [3, 4]. This indicates triple burden of CS — a common scenario for LMICs including Bangladesh. The underlying reasons are unavailability of formal health insurance coverage, higher rate of poverty, and rurality — the issues which are quite significant in Bangladesh [5]. This results in a major lack of access to CS use for many women in LMICs, and Bangladesh in general [5-9], and a few geographical regions (such as Mymensingh, Sylhet, Chattogram) in particular [1, 2, 4, 10, 11]. This contributes to the increasing maternal and child mortality which are challenges in achieving Sustainable Development Goal’s target 3.1 (reduction of maternal mortality), target 3.2 (reduction of under-five mortality) and target 3.7 (ensure universal coverage of sexual and reproductive health) by 2030 [12]. However, the problem has not been addressed by previous research in many LMICs including Bangladesh where rapid rise in CS use has always been the focus of the health researchers and policy makers [6].* | Page 3-4 |
| The objectives of the study were clearly stated as well as its relevance. | We have revised relevant sentences to address these issues. The revised sentences read-  *We aimed to determine the interaction effects of women’s disadvantage characteristics including place of residence, education, wealth quintile, working status on CS use in Bangladesh to fill the existing gap in research.* | Page 4 |
| **Methods** |  |  |
| The "exposure variables" section should be better described. It would be better to have a comprehensive description of the exposure variables and their nature. | We have edited the exposure variables section and provide classification and/or variable creation procedure wherever necessary. Please see the revised section. | Page 5-6 |
| Can the authors indicate whether the sampling weight was used in the descriptive statistics to adjust for the non-proportional distribution of the sample across different regions and their urban and rural areas, as suggested by the DHS procedure | The sampling weights were considered during initial analysis. However, we missed reporting it in our submitted version of the manuscript. We have now added a sentence in the statistical analysis section to specify this issue. The added sentence reads-  *Sampling weights were considered in all analyses* | Page 7 |
| - The use of multilevel modeling is relevant. They specified it by the hierarchical structure of the data. However, they need to make explicit in the variable exposure part, which variables are individual level and which are community level. | We have revised the exposure variable section to clarify variables at the individual, household, and community level. Please see the revised manuscript. Moreover, we have added the following sentences in the statistical analysis section to address the issues of hierarchy broadly-  *In BDHSs, individuals were nested within a household and households were nested within a cluster. Therefore, the samples are hierarchical. i.e they are not independent. For such type of hierarchy, multilevel logistic regression model has been recommended in previous research [13-16]. Therefore, multilevel logistic regression models were used in this study to determine the association of CS use with socio-demographic characteristics at five-time points (BDHS surveys: 2004, 2007, 2011 and 2014).* | Page 6-7 |
| Tell the version of the R software that was used and specify the package that was used to do the multilevel modeling. | We have revised the relevant sentences to address these issues. It now reads-  *The Statistical Package R (version 4.0.3) were used for all statistical analyses. The ‘lme4’ package in R was used for multilevel modelling.* | Page 7 |
| It is assumed that the yearly models have been fitted on the individual and community level variables. However, we have no idea of the variability of the clusters. To do this, we need to give the intra-class correlation coefficient. | Thanks to bring this in our attention. We have now added ICC value for each model. Results of the log likelihood test to provide justification of using multilevel modelling have also provided. Moreover, we have added the following sentences in the results section-  *The results of the multilevel logistic regression model to determine the factors associated with CS use are presented in Table 3. Each model results were compared with their corresponding fixed effect model results though log-likelihood test. The test results were significant for all surveys, which indicate use of multilevel model was appropriate. The cluster level variations of CS use was reported higher for the 2004 survey at around 27%, which was found gradually decreased with the recent surveys and around 11% variability of CS use was reported for the 20-17/18 survey.* | Page 9 |
| Also, the authors need to be more consistent in the presentation of Table 3. Usually the references are the least exposed. Authors choose certain references like middle over other wealth modalities. The notion of reference mode also applies to qualitative explanatory variables. In fact, in a model, all the coefficients are calculated in relation to the reference mode. It is important to choose a reference mode that makes sense in order to facilitate interpretation. Moreover, this choice may also depend on the way in which one wishes to present the results. In general, one should avoid choosing as a reference an item that is poorly represented in the sample or an item that corresponds to an atypical situation. Can the authors specify the reasons for the choice of reference modalities? | We have now presented reference category consistently for each variable. | NA |
| Add the diagram of study flow in the article to help understand the sampling | As suggested, we have added Figure 1 where details sampling procedure are presented. | NA |
| Figure 2: The authors list region of residence among the socioeconomic factors. Unless we describe the regions and highlight their socioeconomic differences, we suggest removing them from this figure | As suggested, we have deleted the figure that was specific for regions. | NA |
| Figure 2: (c) the part "engaged in formal worked/not engaged in formal worked" is not part of the authors' analysis models and therefore should not appear in this work. On the other hand, the status (worked: yes/no) is part of the model and could therefore be included. | Thanks to bring this in our attention. They are basically same with different terms used to define. We have now revised these terms throughout the manuscript to make it consistent. | NA |
| **Discussion** |  |  |
| The discussion is well conducted and explains the reasons for the inequity of access to cesarean section in Bangladesh although some assertions about Bangladesh would need to be supported by references for example: “Private health facilities in Bangladesh are mostly profit ambitious and located in urban centre”. | We have added reference whenever necessary. | NA |
| **Conclusion** |  |  |
| The authors should also show among the perspectives the necessity to make an assessment concerning the indications of caesarean sections. | We have now revised the relevant sentence to address this issue. The sentence now reads-  *Therefore, government programs and regulations are strictly required in order to determine the need for CS as well as to control CS use in general and private health facilities in particular.* | Page 14 |
| **Reviewer#3** |  |  |
| It is a cross-sectional cohort study. The authors establish the association of the social characteristics of women about the use of cesarean section (CS) in Bangladesh; from a multi-stage survey, they were determined in the periods of 2004, 2007, 2011, 2014 and 2017/18, The analysis is under what is proposed in general. In general terms, well written that part of a valid research question, and it is possible to establish how sociodemographic differences affect the use of the cesarean section, increasing inequalities. |  |  |
| I want the authors to clarify the hierarchical levels that were taken into account for the multilevel analysis. | This issue has already been addressed in responding the second reviewer’s comment. We have revised the exposure variable section to clarify variables at the individual, household, and community level. Please see the revised manuscript. Moreover, we have added the following sentences in the statistical analysis section to address the issues of hierarchy broadly-  *In BDHSs, individuals were nested within a household and households were nested within a cluster. Therefore, the samples are hierarchical. i.e they are not independent. For such type of hierarchy, multilevel logistic regression model has been recommended in previous research [13-16]. Therefore, multilevel logistic regression models were used in this study to determine the association of CS use with socio-demographic characteristics at five-time points (BDHS surveys: 2004, 2007, 2011 and 2014).* | Page 6-7 |
